# Impact of different types of green manure on pests and disease incidence and severity as well as growth and yield parameters of maize

**DOI:** 10.1016/j.heliyon.2023.e17294

**Published:** 2023-06-15

**Authors:** Francess Sia Saquee, Prince Emmanuel Norman, Musa Decius Saffa, Nyasha John Kavhiza, Elena Pakina, Meisam Zargar, Simbo Diakite, Gani Stybayev, Aliya Baitelenova, Gulden Kipshakbayeva

**Affiliations:** a Department of Agrobiotechnology, Institute of Agriculture, RUDN University, 117198, Moscow, Russia; b Faculty of Development Agriculture & Natural Resources Management, Eastern Technical University of Sierra Leone, Combema Road, Kenema City, 00232, Sierra Leone; c Sierra Leone Agricultural Research Institute (SLARI), PMB 1313, Tower Hill, Freetown, Sierra Leone; d Njala University, School of Agriculture and Food Sciences, Crop Protection Department, Sierra Leone; e Department of Plant Protection, Faculty of Agronomy, S. Seifullin Kazakh Agrotechnical University, 010000, Astana, Kazakhstan

**Keywords:** *Calopogonium*-*Pueraria* mixture (CAL), *Panicum* (PAN) green manure, *Zea mays*, Pest, Disease

## Abstract

The emergence of pests and diseases, including the maize streak virus, leaf blight, the African stem borer, and gray leaf spot, poses a persistent threat to maize production (*Zea mays* L. cv: DMR-ESR-Yellow) around the world. A field experiment was conducted at the School of Agriculture experimental site, Njala University, Sierra Leone, during a two-year period (2020–2021) to assess the effects of green manure on pest and disease incidence and severity as well as growth and yield parameters of maize. The experiment was laid out in a randomized complete block design (RCBD) with three replications and four treatments: Cal. 3 t.ha^−1^, Cal. 6 t.h^−1^, Pan. 3 t.h^−1^, Pan 6 t.ha^−1^ and a control plot amended with 200 kg ha^−1^ of N (urea) and NPK 15:15:15 ha^−1^ split application. The study showed that gray leaf spot damage was the most severe infection among all treatments. Therefore, the effects of the most severe disease and pest of maize in Sierra Leone can be minimized by applying green manure. Moreover, results reveal that *Calopogonium- Pueraria* mixture amended plots showed significant performance in the measured growth parameters viz. highest leaf number, large leaf area stem girth, superior plant height, best ear height (64.6–78.5 cm), higher cob yield (1.2–1.4 t.ha^−1^) ear (1.8–2.1 t.ha^−1^) and dry grain yield (0.5–0.7 ha^−1^). *Panicum* green manure results showed that prompt and adequate application, as well as decomposition of green manures, is imperative for the successful conservation and sustainability of maize farming systems. The findings of this research could improve the efficiency of green manure use in pest, disease, and crop management systems.

## 1 Introduction

Maize (*Zea mays* L.) belongs to the grass family (Poaceae) and originated from Central America [1]. The crop was first introduced in Africa by the Portuguese at the beginning of the 16th century [2]. The maize plant has a fibrous root system that spreads in the soil and has a solid unbranched stem [3]. The plant has long, narrow leaves and a cob producing many grains with high starch content. The mature plant bears a tassel at its tip. Maize is a major crop grown in many regions of the world and a staple diet for a significant portion of the world’s population especially those in developing nations, including Sierra Leone. This crop has high nutrient requirements, especially for nitrogen, and is termed a “Heavy feeder”, due to its voracious affinity for nutrients. The requirement to produce enough food to support the fast-expanding global population, minimize hunger among people, and supply adequate livestock feed is a challenge for Africa, particularly in Sierra Leone. As a result of exponential population growth, there is a surging demand for cereal crops to supplement root and tuber crops. Maize accounts for 20% of the calories consumed by the world’s populace, directly or indirectly [4].

Even though maize has a wide range of uses, only 2.1% of Sierra Leonean households are engaged in its farming [5]. The causes seem multifaceted: the pressure of diseases and pests in particular, lack of organization in the agricultural sector, the lack of high-quality genetic material, the high cost of fertilizers, climate change, and the lack of proper agronomic practices. According to Pavan and Shete [6], the corn crop is constantly attacked by insects and over 65 diseases, such as fungi, bacteria, and viruses, throughout its production cycle. Among these diseases and pests, maize streak virus (MSV), leaf blight (Helminthosporium turcicum), gray leaf spot (*Cercospora zeae-maydis*), and African stem borer (*Busseola fusca*), pose a persistent threat to maize production in Sierra Leone. More than 54% of production losses in this crop are attributed to diseases and insect pests. In terms of ranking, diseases are the second most crucial factor affecting the quality and productivity of corn after insects [7].

Several methods commonly used to mitigate losses caused by these organisms have limitations. For example, nitrogen fertilizers have been considered an imperative means to increase yield for several decades; however, they are also often inappropriate in specific scenarios due to their (high cost, destruction of soil microfauna, etc.). Also, with the use of pesticides, herbicides, and fungicides, the environment will suffer short and long-term effects, including increased soil porosity leading to erosion, decreased soil organic carbon, leaching of gaseous emission hence depleting soil fertility and soil organic matter levels as well as reduced carbon dioxide sequestration [8]. It is, therefore, urgent to develop fertilization techniques and regimes that are more easily accessible to farmers and environmentally friendly. Among other possible solutions, herbaceous, perennial, or subligneous legumes such as Calopogonium, Centrosema, Clitoria, Crotalaria, *Panicum*, *Pueraria,* etc., can be used. Therefore, green manures can be applied the maize crop to reduce the incidence and severity of pests and diseases, thus improving yields [7,9].

Green manure is a predecessor crop grown specifically to improve the soil and supply nutrients for successive crops. It can raise soil organic matter, control pests and diseases, and increase crop yield [10]. According to Baiyeri and Olajide [11], it was demonstrated that using green manure helps manage agricultural pests and diseases, minimize erosion, control weeds, and provide habitat for beneficial species in addition to improving soil health and fertility. Emphasis is put on the practical significance of green manuring in intensifying sustainable crop production systems. Given that they are considered plants that improve soil, green manure is a practical and sustainable solution to minimize the incidence and severity of disease and pests and increase yield. Green manure can also mineralize organic matter, boost root exudates, amino acids, and plant hormones, among other substances, and recycle nutrients for the succeeding crop [12]. Furthermore, allelopathy, which involves the release of secondary metabolites by leaves, decomposing straw, or the root system, may also aid in reducing weed populations [13].

There are still knowledge gaps on the efficacy of green manure in pest and disease control and its consequent effects on crop yield. This research explored the following green manures, *Calopogonium* spp.*, Pueraria* spp.*, Panicum* spp. They have a symbiotic relationship with certain soil bacteria; these bacteria form nodules on the roots and fix atmospheric nitrogen. Some of this nitrogen is utilized by growing plants, but some can also be used by other plants growing in proximity [14]. Calopogonium has specific rhizobium requirements for rhizobium and nodulates readily. It provides soil N to neighboring grasses when it is sown in mixed stands, with tall tropical companion grasses such as *Panicum* spp., and when intercropped (with maize, for instance). Its effects can last 14–16 years in the soil [15].

*Pueraria* spp. is a productive cover crop and green-manure plant (often intercropped with other leguminous species such *as Calopogonium* and *Centrosema*). It is grown amongst various plantation crops (rubber, oil palm, coffee, cocoa, citrus). The plant is tolerant and better adapted to tropical lowlands and uplands. When grown with other legume species, it suppresses weed infestation, controls erosion on hilly slopes, enriches the soil by fixation of atmospheric nitrogen by the root nodules, and adds organic matter from its leaf litter. It is an N-fixing legume and serves as an excellent soil cover. Horrell [16] states that the plant can also be used for mulching, hence adding considerable nitrogen by mineralizing leaf litter.

**Fig. 1:**
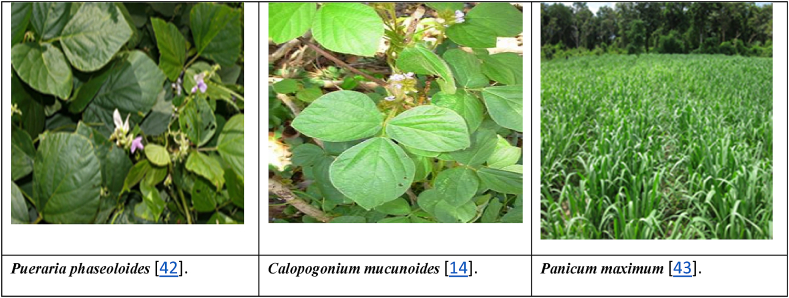
Various green manures were used in this experiment [14,42,43]. (For interpretation of the references to colour in this figure legend, the reader is referred to the Web version of this article.)

*Panicum* spp. is a warm-season perennial C4 grass native to North America, where it denotes a conventional variety in the tall grass plains [17]. The accepted use of this plant was related to soil conservation and fodder production [18]. *Panicum maximum* is a fast-growing, bulky grass that helps prevent soil erosion since it supplies rapid ground cover (Fig. 1) [19].

Researchers have examined a few dynamics to demonstrate how maize yields differ from one part of Africa to another. To find places where yields may have stagnated, the researchers conducted a global, regional, and national analysis of maize production patterns using green manuring to control pests and diseases of maize [[20], [21], [22], [23], [24], [25]]. Furthermore, other studies have extensively investigated the use of green manure in crop production for a variety of crops, including rice [26,27], maize [28,29], okra [30], tomatoes [31], and wheat [32]. In addition to boosting crop production, it also decreases the use of chemical fertilizers, consequently increasing soil microbial activity [[32], [33], [34], [35]]. Moreover, green manure improves the soil’s biological and chemical characteristics of soil, hence increasing the diversity of soil microbes [[36], [37], [38]].

However, a more geographically comprehensive assessment of the trends in maize yield, disease, and pest incidence and severity, and yield harvested area has to be fully attempted in Africa, especially in Sierra Leone. The only attempt to investigate maize yield harvest area dynamics is based on a study by Ref. [39], which examines the drivers of maize yield expansion in sub-Saharan Africa and policies to improve maize production. Research on the use of green manure, such as *Calopogonium*-*Pueraria* mixture and *Panicium,* on the incidence, severity, and growth of maize is still lacking, thus it was interesting to study the best variables for using these green manuring to suppress the incidence and severity of pests and diseases of maize over time and examine the growth and yield parameters of maize.

## 2 Materials and methods

### 2.1 Study area and site

This study was conducted in the upland at Njala University Campus-Njala Agriculture site in the Kori Chiefdom Moyamba District. The experimental site is located at an elevation of 50 m above sea level on 8° 06N latitude and 12°06W longitude. Njala has two distinct seasons, the wet season (May to October) and the dry season (November to April). Average annual precipitation ranges from 2125 to 2526 mm, and the maximum temperature ranges from 29° to 34 °C, while the minimum air temperature ranges from 21° to 23 °C. The relative humidity is very high, close to 100% for the more significant part of the day and night, especially during the rainy season [40]. During the dry season, potential evapotranspiration exceeds rainfall, while during the rainy season, precipitation exceeds evapotranspiration. Due to continuous farming and other economic activities, the predominant vegetation is secondary farm bush grassland. The moderately fertile soils have a balanced mixture of sand, clay, and humus. The dominant soil type was the Njala series (orthotic palahumult). The soil moisture varies during the dry season, has low nutrient status, and is slightly acidic, with a pH ranging from 5.5 to 6.0.

### 2.2 Soil sample analysis

The soil sample was conducted to determine the nutrient status of the experimental site before planting (Table 1). A composite soil sample was obtained by thoroughly mixing subsamples collected from randomly selected points within the field at a depth of 01–20 cm. The soil sample was taken to the Sierra Leone Agricultural Research Institute (SLARI) laboratory and analyzed.

**Table 1:** Physicochemical characteristics of the soils used for the field trials.

Soil parameters	Value
Bulk density (g cm^−3^)	1.80
Soil Ph	3.91
Organic matter (%)	1.84
Total N (%)	0.13
Available P (μg g^−1^)	8.56
Exchangeable bases (cmolc kg^−1^)	0.00
Ca^2+^	0.14
Mg^2+^	0.11
K^+^	0.08
Na^+^	0.15
Exchangeable acidity (cmolc kg^−1^)	2.40
CEC (cmolc kg^−1^)	9.82
Sandy (%)	83.01
Silt (%)	6.23
Clay (%)	11.24
Textural class	Sandy loam

### 2.3 Experimental design and land preparation

The experiment was carried out in a period of 2 years i.e. 2020 and 2021, the experiment was arranged in a randomized complete block design (RCBD) with three replications. The treatments include: (1) 6 kg *Calopogonium*-*Pueraria* mixture (T1); (2) 3 kg *Calopogo*nium- *Pueraria* mixture (T2); (3) 6 kg *Panicium* (T3); (4) 3 kg *Panicium* (T4); and (5) Fertilizer application (NPK and Urea) served as control amended with 200 kgN ha^−1^ (urea) and NPK 15:15:15 ha^−1^ split application. The dimensions of the experimental field were 27 m × 11 m. In each experimental year, from June to August (traditional maize growing season in Sierra Leone) standard agricultural practices such as land preparation, planting, weeding and harvesting were performed. Fertilizer application was carried out for the control in both seasons 2020 and 2021. Seventy-two planting ridges were constructed translating to four planting ridges per plot. Each plot measured 3 m × 5 m with a spacing of 0.5 m between plots and 1 m between two adjacent blocks. Each block/replication consisted of 6 plots, making a total of 18 plots. Each planting ridge had 18 plants, giving 72 plants per plot and 432 plants per replication.

The ridges were incorporated with the two different types of green manure weighed per treatment (3 t.ha^−1^ and 6 t.ha^−1^ of *Calopogonium*-*Pueraria* mixture and *Panicum* green manure, respectively); these were incorporated into the ridges and then left to decompose for 2 weeks before planting. A 90-day maize variety “Western Yellow” was used in this study. The planting distance was 75 cm between rows and 50 cm within rows. After emergence, the seedlings were thinned to two plants per stand, resulting in 1296 plants. The split fertilizer application method was adopted for control. To attain the different fertilizer application rates, NPK (15:15.15) was used for basal application and supplemented with Urea (46% N) and were applied at four weeks and six weeks after planting in a band about 5 cm away from the plant stands to a depth of 5 cm respectively in all control plots. Weeding was done for 3 and 8 weeks after planting. No insecticides were applied to the plants.

### 2.4 Data collection

Data collection was done in all 18 plots during the cropping season. On each plot, 20 plants from the middle rows were randomly selected and tagged for data collection on pest, diseases, growth, and yield parameters, discarding the two extreme plants on both ends of each ridge sampled. All growth parameters, pest and disease incidence, and severity were measured and recorded four weeks after emergence (WAE), followed by biweekly data recordings (Table 2). Yield data was collected at physiological maturity. Plant height was measured and recorded four weeks after emergence, followed by biweekly data recordings at 50% days to tasseling. Plant height is the average height of plants in centimeters (cm) from the base of the plant to where tassel branching begins, using a meter rule. All other yield parameters (number of leaves per plant, leaf area, stem girth, days to 50% tassel, ear height (cm), fresh cob yield, fresh ear yield, dry grain yield, and 1000 seed weight) were recorded. Grain yield was determined by measuring the total weight of maize per plot at 12.5% moisture content with a balance and expressed in tons per hectare. Pest and disease incidence and severity were ascertained by visually examining and recording the number of maize plants showing the disease and pest symptoms, and the percentage incidence was calculated as follows:

$$Incidence\;\left(\%\right)=\frac{Number\;of\;infected\;plants}{Number\;of\;plants\;assessed}\times 100$$

**Table 2:** Visual rating scale used for four diseases and one pest severity found in the study field.

Rating scale	Description	Expression in terms of severity
1	No symptoms No infection	No infection
2	Very few signs and symptoms on leaves	Mild
3	Moderate signs and symptoms of old and young leaves, slight stunting	Moderate infection
4	Severe signs and symptoms about 60–75% of leaf area, plants stunted	Severe infection
5	Severe signs and symptoms on more than 75% of leaf area	Very severe infection severely stunted or dead plants

Plants scored for disease severity placed on a range of 1–5 as implemented by Bosque-Pérez and Alam [41].

### 2.5 Data analysis

All the data collected were subjected to analysis of variance (ANOVA) using the GENSTAT statistical program (GENSTAT, 15th release, Rothampstead, UK). The Least Significance Difference (LSD) was used to compare treatment means using a significance level of α = 0.05. The residuals of the parameters studied were first checked for normality and homogeneity using the Shapiro-Wilk and Bartlett’s tests to ensure that data were normally distributed.

## 3 Results and discussion

### 3.1 Incidence and severity of various diseases and pests

Combined ANOVA indicated no statistical differences between year and experimental treatments on measured traits. Generally, leaf blight incidence and severity significantly (p < 0.001) increased with time among treatments (Table 3). Four weeks after emergence (WAE), the PAN 6 t.ha^−1^ amended, and control plots had no visible attack of the disease, whilst the remaining treatments had a mild infection. Both treatments PAN 6 t.ha^−1^ and control consistently exhibited mild disease attack up to 10 WAE, whereas the remaining treatments exhibited persistent attack, with disease scores ranging between 3.6 and 4.0 at the 10 WAE sampling regime.

**Table 3:** Effects of green manures and rates on mean leaf blight incidence, for cropping season 2020–2021.

	Leaf blight severity				Leaf blight incidence (%)			
	Weeks after emergence				Weeks after emergence			
Treatments	4	6	8	10	4	6	8	10
CAL 6 t.ha^−1^	1.7	2.7	3.4	3.6	16.7	44.4	66.6	72.2
CAL 3 t.ha^−1^	2.3	3.3	3.0	3.8	44.4	55.5	61.1	83.3
PAN 6 t.ha^−1^	1.0	3.2	2.4	2.7	0.0	27.8	44.4	44.4
PAN 3 t.ha^−1^	1.7	3.2	3.9	4.0	11.1	38.9	61.12	72.2
CONTROL	1.0	1.0	1.6	1.8	0.0	0.0	22.2	22.2
MEAN	1.5	2.5	2.9	3.2	14.4	33.3	51.1	58.8
LSD Treat (5%)	0.8***				20.0***			
LSD Samreg (5%)	0.7***				17.9***			
CV%	12.5				29.3			

LSD Treat = least significant difference between the treatments; LSD Samreg = least significant difference between the sampling regimes; *** = significant at p < 0.001; CAL (Calopogonium-pueraria mixture), PAN (Panicum), and Control, amended with NPK 15:15:15 at 200 kg.Nha^−1^.

The Maize Streak Virus (MSV) severity and incidence percentage significantly varied among treatments. Treatments CAL 6 t.ha^−1^, PAN 3 t.ha^−1^ and control had no visible disease symptoms, whilst CAL 3 t.ha^−1^ and PAN 6 t ha^−1^ had a mild disease attack with 27.8% incidence at 10 WAE (Table 4).

**Table 4:** Effects of green manure and rates on maize streak virus (MSV) severity and incidence.

	MSV severity				MSV incidence (%)			
	Weeks after emergence				Weeks after emergence			
Treatments	4	6	8	10	4	6	8	10
CAL 6 t.ha^−1^	1.0	1.0	1.0	1.0	0.0	0.0	0.0	0.0
CAL 3 t.ha^−1^	1.7	1.5	1.7	1.8	11.1	16.7	27.8	27.8
PAN 6 t.ha^−1^	2.0	2.0	1.7	1.9	5.5	5.0	16.7	27.8
PAN 3 t.ha^−1^	1.0	1.0	1.0	1.0	0.0	0.0	0.0	0.0
CONTROL	1.0	1.0	1.0	1.0	0.0	0.0	0.0	0.0
MEAN	1.3	1.3	1.3	1.3	3.4	4.5	8.9	11.1
LSD Treat (5%)	0.6**				15.0**			
LSD Samreg (5%)	0.5^ns^				13.4*			
CV%	41.0				172.2			

LSD Treat = least significant difference between the treatments; LSD Samreg = least significant difference between the sampling regimes; ns = not significant; * = significant at p < 0.05; ** = significant at p < 0.01; CAL (*Calopogonium*-*pueraria* mixture), PAN (*Panicum*), and Control, amended with NPK 15:15:15 at 200 kg.Nha^−1^.

Gray Leaf spot severity and incidence significantly increased with time among treatments (Table 5). At 4 and 6 WAE, the green manure and control treatments showed low attack of the disease, whilst, at 8 and 10 WAE, most of the treatments, including the control, showed intermediate attack of the disease. Plots amended with PAN 3 t.ha^−1^ showed the highest attack of the disease (4.7), followed by CAL 6 t.ha^−1^ (4.4).

**Table 5:** Effects of green manure and rates on the severity of gray leaf spot on maize.

	Leaf spot severity				Leaf spot incidence (%)			
	Weeks after emergence				Weeks after emergence			
Treatments	4	6	8	10	4	6	8	10
CAL 6 t.ha^−1^	3.2	3.9	4.2	4.4	100	100	100	100
CAL 3 t.ha^−1^	3.2	3.7	4.1	3.9	100	100	100	100
PAN 6 t.ha^−1^	3.3	3.7	3.9	4.2	100	100	100	100
PAN 3 t.ha^−1^	3.0	3.5	4.3	4.7	100	100	100	100
CONTROL	2.9	3.2	3.7	4.0	100	100	100	100
MEAN	3.1	3.6	4.0	4.3	100	100	100	100
LSD Treat (5%)	0.3***				0.0^ns^			
LSD Samreg (5%)	0.2***				0.0^ns^			
CV (%)	3.6				0.0			

LSD Treat = least significant difference between the treatments; LSD Samreg = least significant difference between the sampling regimes; ns = not significant; *** = significant at p < 0.001; CAL (*Calopogonium*-*Pueraria* mixture), PAN (Panicum), and Control, amended with NPK 15:15:15 at 200 kg.Nha^−1^.

The mean on Africa Stem Borer severity and percent incidence significantly varied among treatments at 8 and 10 WAE (Table 6). All green manure treatments showed no visible disease symptoms during 4, 6 and 8 WAE as compared to the control. At 10 WAE, PAN 3 t.ha^−1^ showed no visible disease expression, while others had a mild attack. The disease incidence followed a similar trend, with the control displaying the highest attack of 38.9% as compared to the green manure amended plots. The finding shows a visible invasion of African stem borer was only observed in the green manure amended plots at 10 WAE, whereas PAN 3 t. ha^−1^ exhibited no symptoms of the disease throughout the sampling regimes.

**Table 6:** Effects of green manures and rates on mean African Stem Borer (ASB) severity and incidence.

	ASB severity				ASB incidence (%)			
	Weeks after emergence				Weeks after emergence			
Treatments	4	6	8	10	4	6	8	10
CAL 6 t.ha^−1^	1.0	1.0	1.0	1.5	0.0	0.0	0.0	16.7
CAL 3 t.ha^−1^	1.0	1.0	1.0	1.9	0.0	0.0	0.0	27.8
PAN 6 t.ha^−1^	1.0	1.0	1.0	1.2	0.0	0.0	0.0	11.1
PAN 3 t.ha^−1^	1.0	1.0	1.0	1.0	0.0	0.0	0.0	0.0
CONTROL	1.0	1.0	1.8	2.1	0.0	0.0	16.7	38.9
MEAN	1.0	1.0	1.2	1.5	0.0	0.0	3.3	18.9
LDS Treat (5%)	0.4**				13.0**			
LDS Samreg (5%)	0.3**				11.6**			
CV (%)	11.6				42.8			

LSD Treat = least significant difference between the treatments; LSD Samreg = least significant difference between the sampling regimes; CV = coefficient of variation; ** = significant at p < 0.01; CAL (*Calopogonium*-*Pueraria* mixture), PAN (*Panicum*), and Control, amended with NPK 15:15:15 at 200 kg.Nha^−1^.

### 3.2 Effects of green manure and rates on the growth of maize parameters (leaf number and leaf area (cm^2^)

*Calopogonium*-mixture amended plants significantly (p < 0.001) produced the highest leaf number and largest leaf area compared to *Panicum* manure during the 4–8 WAE sampling regimes (Table 7). Leaf number increased with increasing application of *Calopogonium*-mixture as decomposition continued, while plants amended with panicum consistently showed fewer leaves than the control, this might be due to the slow decomposition rate of *Panicum* green manure.

**Table 7:** Effects of green manure and rates on the growth of maize.

	Number of leaves per plant			Leaf area (cm^2^)		
	Weeks after planting			Weeks after planting		
Treatments	4	6	8	4	6	8
CAL 6 t.ha^−1^	7.7	9.4	13.2	287	547	472
CAL 3 t.ha^−1^	6.8	8.5	12.4	258	385	324
PAN 6 t.ha^−1^	6.4	7.7	11.7	126	374	383
PAN 3 t.ha^−1^	6.4	7.1	11.8	143	350	336
CONTROL	7.4	8.7	11.1	183	533	683
MEAN	7.1	8.3	12.4	199	438	440
LSD Treat (5%)	0.6***			86.9***		
LSD Samreg (5%)	0.5***			67.3***		
CV (%)	2.0			9.7		

LSD Treat = least significant difference between the treatments; LSD Samreg = least significant difference between the sampling regimes; CV = coefficient of variation; *** = significant at p < 0.001; CAL (*Calopogonium*-*Pueraria* mixture), PAN (*Panicum*), and Control, amended with NPK 15:15:15 at 200 kg.Nha^−1^.

### 3.3 Effects of green manure and rates on plant height (cm) and stem girth (cm)

Both green manure types and rates had significant (p < 0.001) effects on plant height and stem diameter (Table 8). Generally, plant height and stem diameter increased as the sampling regime progressed from 4 to 10 WAE. Besides the control, CAL 6 t. ha^−1^ significantly showed higher plant height (51.2 cm and 128.4 cm) as well as wider diameter (5.5 cm and 6.5 cm), respectively, at 6 and 8 sampling regimes, while panicum amended plots exhibited the lowest (31.1 cm and 92.0 cm; 5 cm and 5 cm).

**Table 8:** Effects of green manures and rates on mean plant height (cm) and stem diameter (cm).

	Plant height (cm)			Stem diameter (cm)		
	Weeks after planting			Weeks after planting		
Treatments	4	6	8	4	6	8
CAL 6 t.ha^−1^	19.4	51.2	128.4	5.4	5.5	6.5
CAL 3 t.ha^−1^	14.8	40.9	112.9	4.0	5.2	5.7
PAN 6 t.ha^−1^	12.4	31.7	99.9	3.8	5.3	5.4
PAN 3 t.ha^−1^	12.0	31.1	92.0	3.6	5.0	5.0
CONTROL	13.8	44.8	145.6	4.9	6.5	6.6
MEAN	14.5	40.0	115.8	4.3	5.5	5.9
LSD Treat (5%)	12.1***			0.4***		
LSD Samreg (5%)	9.4***			0.3***		
CV (%)	6.2			6.5		

LSD Treat = least significant difference between the treatments; LSD Samreg = least significant difference between the sampling regimes; CV = coefficient of variation; *** = significant at p < 0.001; CAL (Calopogonium-pueraria mixture), PAN (Panicum), and Control, amended with NPK 15:15:15 at 200 kg.Nha^−1^.

### 3.4 Effects of green manures and rates on yield and yield attributes of maize

The two types of manure and rate had a significant (p < 0.001) effect on days to 50% tassel, yield, and yield attributes of maize (Table 9). Application of panicum at 6 t. ha^−1^ took a significantly extended period to reach 50% tasseling compared to the remaining treatments. The fresh and dry yields of *Calopogonium* amended plants significantly outyielded panicum at 6 t.ha^−1^ rate, while the control had the highest fresh cob (2.889 t.ha^−1^), followed by CAL 6 t.h^−1^ (1.422 t.h^−1^), and the control plot had dry grain yield reaching (1.044 t.ha^−1^), and 1000 seed weigh of (146.8 g), followed by CAL 6 t.ha^−1^ (158.8 g).

**Table 9:** Effects of green manure and rates on days to 50% tasseling, ear height (cm), fresh cob yield (t.ha^−1^), fresh ear yield (t.ha^−1^), dry grain yield (t.ha^−1^) and 1000- seed weight (g).

Treatments	Days to 50% tassel	Ear height (cm)	Fresh cob yield (t. ha^−1^)	Fresh ear yield (t. ha^−1^)	Dry grain yield (t. ha ^−1^)	1000 seed weight (g)
CAL 6 t.ha^−1^	62.67	78.5	1.422	2.089	0.667	158.0
CAL 3 t.ha^−1^	62.67	64.4	1.156	1.756	0.467	145.9
PAN 6 t.ha^−1^	65.67	51.7	0.844	1.400	0.333	151.7
PAN 3 t.ha^−1^	62.67	54.8	0.822	1.422	0.489	154.9
CONTROL	64.67	85.2	2.889	4.622	1.044	146.8
MEAN	64.67	66.9	1.427	2.258	0.600	151.5
LSD Treat (5%)	2.42**	13.1***	0.507***	0.551***	0.293**	23.7^ns^
CV (%)	1.6	5.8	35.9	31.4	28.9	5.8

LSD Treat = least significant difference between the treatments; CV = coefficient of variation; ns = not significant; ** = significant at p < 0.01; *** = significant at p < 0.001; CAL *Calopogonium-pueraria* mixture; PAN *Panicum*, CONTROL, amended with NPK 15:15:15 at 200 kg.Nha^−1^.

## 4 Discussion

Leaf blight incidence exhibited a similar trend as disease severity. Leaf blight lesions have been noted to reduce the leaf area of maize, thereby limiting the process of photosynthesis. The earlier the lesions develop, the higher the spread of the lesions and the greater the damage to the crop photosynthetic area. Mueller et al. [44] reported that up to 30% of yield loss had been reported in hybrid corn severely infected before or at tasseling. Similarly, the increasingly severe attack of the disease may significantly contribute to low yields obtained. The control plots registered a mild disease attack, followed by 3 t.ha^−1^ application of *Calopogonium*-mixture and 3 t.ha^−1^ *Panicum*.

According to Guthrie [45] and Bosque-Pérez et al. [46], when Chlorotic striping progresses to chlorosis of the entire lamina in extremely sensitive types, and MSV infection occurs at an early stage of plant development, this could be followed by progressive necrosis and plant death, hence inhibiting maize development and in susceptible varieties, yield reductions frequently exceed 70%. Minimal yield loss occurs if an infection occurs after eight weeks of emergence. A yield loss of 1–5% caused by MSV has often been observed in the Eastern and Southern African regions [47]. In the present study, MSV infection was mild throughout the sampling regimes. Thus, an attack by this disease was not significant.

Gray leaf spot severity on maize may have contributed to decreasing yields obtained in this study. These findings confirmed an earlier report by Bhatia and Munkvold [48] that yield loss by gray leaf spot depends on the extent of lesion damage in the canopy during tasseling and pollination phases. The earlier lesion development in this study may have contributed to yield loss since symptoms of the disease observed reached the ear leaf and were higher during the two weeks before and after tasseling. The reduced leaf number and leaf area were probably related to the varying nutrient release pattern of the green manure types.

Findings on African stem borer incidence and severity indicated slight visible attack on all green manure amended plots at 10 WAE, with PAN 3 t.ha^−1^ exhibiting mild signs of attack throughout the sampling regimes. The observations from this study are congruent with those of De Groote [49] stating a 13.5% yield loss caused by stem borers in Kenya, which translates to 400,000 tons of maize each year. The variance may possibly be due to differences in varieties used and climate conditions in which they were grown.

The plant height and stem diameter difference might be due to an initial N immobilization in the panicum amended plot. Findings indicate that panicum green manure may need to release more initial nitrogen to support early growth. Similarly, Olfs et al. [50] observed nitrogen as the most critical plant nutrient limiting crop growth.

Generally, plants amended with higher rates of green manure and the control consistently produced higher yields and yield components compared to the lower application rates. Results agree with Csizinsky [51], who also noted a linear increase in fresh yields as the N rate increased. The increasing rates of manures used in this study may have increased the organic matter content and available soil nitrogen, enhancing its uptake and utilization by the plant. The application of organic manure increased maize growth and yield parameters, including the number of days to tasseling. Results obtained by Arantes et al. [52] demonstrate that *Calopogonium*, when intercropped with maize and sown at the fourth expanded maize leaf stage, produces the highest amounts of dry matter and soil coverage as well as higher final plant stand than maize monoculture. They further stated that the plots of *Calopogonium*, tropical kudzu (*Panicum*), and perennial soy intercropped with maize do not affect the maize grain yield in the organic system; this may be due to the slow decomposition and release of the Panicum incorporated.

## 5 Conclusion

This study established that types and rates of green manure application influence pest and disease incidence and severity as well as growth and yield parameters of maize. *Calopogonium*-*Pueraria* (CAL) mixture amended plots enhanced growth and yield parameters more than *Panicum* (PAN) amended plots that could be exploited for sustainable productivity of the crop. CAL 6 t. ha^−1^ amended plants exhibited higher growth (number of leaves, leaf area, plant height and stem diameter), and yield attribute traits (ear height, fresh cob yield, fresh ear yield, dry grain yield and 1000 seed weight) compared to PAN amended plants. Pest and disease incidence and severity increased with time and is influenced by green manure application type. Results suggest that adequate application of a good green manure type is imperative for sustainable maize farming systems.

## Author contribution statement

Francess Sia Saquee: Conceived and designed the experiments; Performed the experiments; Wrote the paper.

Prince Emmanuel Norman: Conceived and designed the experiments; Analyzed and interpreted the data; Wrote the paper.

Musa Decius Saffa: Nyasha John Kavhiza: Performed the experiments; Wrote the paper.

Elena Pakina: Meisam Zargar: Analyzed and interpreted the data.

Simbo Diakite: Gani Stybayev: Aliya Baitelenova: Gulden Kipshakbayeva: Contributed reagents, materials, analysis tools or data.

## Data availability statement

Data will be made available on request.

## Additional information

No additional information is available for this paper.

## Declaration of competing interest

The authors declare that they have no known competing financial interests or personal relationships that could have appeared to influence the work reported in this paper
